# Exploiting Concepts of Instance Segmentation to Boost Detection in Challenging Environments

**DOI:** 10.3390/s22103703

**Published:** 2022-05-12

**Authors:** Khurram Azeem Hashmi, Alain Pagani, Marcus Liwicki, Didier Stricker, Muhammad Zeshan Afzal

**Affiliations:** 1 Department of Computer Science, Technical University of Kaiserslautern, 67663 Kaiserslautern, Germany; didier.stricker@dfki.de (D.S.); muhammad_zeshan.afzal@dfki.de (M.Z.A.); 2 Mindgarage, Technical University of Kaiserslautern, 67663 Kaiserslautern, Germany; 3 German Research Institute for Artificial Intelligence (DFKI), 67663 Kaiserslautern, Germany; alain.pagani@dfki.de; 4 Department of Computer Science, Lulea University of Technology, 971 87 Lulea, Sweden; marcus.liwicki@ltu.se

**Keywords:** object detection, challenging environments, low-light, complex environments, deep neural networks, computer vision

## Abstract

In recent years, due to the advancements in machine learning, object detection has become a mainstream task in the computer vision domain. The first phase of object detection is to find the regions where objects can exist. With the improvements in deep learning, traditional approaches, such as sliding windows and manual feature selection techniques, have been replaced with deep learning techniques. However, object detection algorithms face a problem when performed in low light, challenging weather, and crowded scenes, similar to any other task. Such an environment is termed a challenging environment. This paper exploits pixel-level information to improve detection under challenging situations. To this end, we exploit the recently proposed hybrid task cascade network. This network works collaboratively with detection and segmentation heads at different cascade levels. We evaluate the proposed methods on three complex datasets of ExDark, CURE-TSD, and RESIDE, and achieve a mAP of 0.71, 0.52, and 0.43, respectively. Our experimental results assert the efficacy of the proposed approach.

## 1. Introduction

One of the most important and widely used tasks in the field of computer vision is object detection. Over the years, many techniques have been employed to improve the performance of object detection. Object detection has various applications, such as instance segmentation [1,2,3], visual question answering [4], image captioning [5,6], object tracking [7], activity recognition [8,9,10], and so on. The process of object detection can be broken down into the following steps: identifying the object and spatial localization of the object to provide exact coordinates of the object’s location.

**Figure 1:**
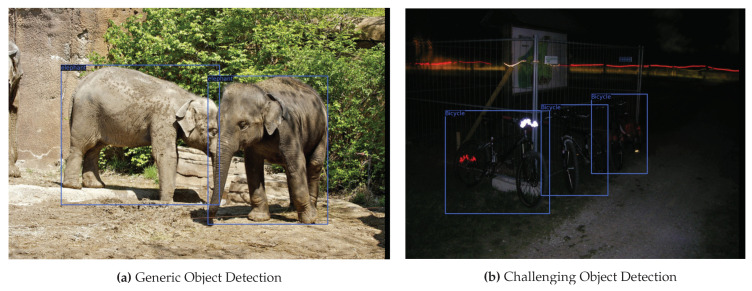
Visual illustration of the difference between object detection in a generic and challenging environment. (**a**) A sample image taken from the COCO dataset [12], whereas (**b**) is taken from the ExDark dataset [13]. The blue color represents ground truth annotation.

Object detection algorithm environments can mainly be categorized into two types [11], object detection in a general environment and object detection in a challenging environment. A general environment is rich in contextual features and has low object cluttering and occlusions. Compared to the general environment, a challenging environment is composed of low contextual features, object cluttering, various occlusions, and objects merged with the background. In real-time scenarios, the input images received by the object detection network are (frequently) not spatially rich as they are captured in complex scenarios and have low-light conditions. In this paper, we have referred all these situations to a challenging environment. Figure 1 illustrates the difference between generic and challenging object detection.

Recently, various approaches, such as a fusion of domains using glue layers [14], fusing thermal images with RGB images [15], and a combination [16] of deep convolution generative adversarial network(s) (DCGAN) [17] and faster R-CNN [18] have been proposed to tackle the problem of object detection in challenging environments. These approaches improved the performance, but are dependent on image enhancement as a pre-processing step and prior assumptions about the type and shape of objects.

**Figure 2:**
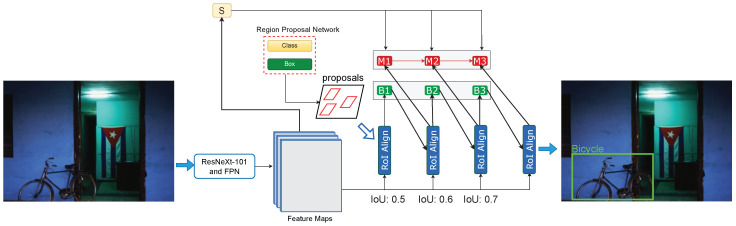
Illustration of the proposed framework. The combination of ResNext-101 and feature pyramid network (FPN) extracts the spatial features from the input image on various scales. The features are propagated to the region proposal network to generate candidate regions. The cascaded R-CNN block further refines the bounding box and masks prediction by leveraging semantic features in the second stage. The optimized proposal features from the last stage are propagated to compute final predictions.

Ahmed et al. [11] recently investigated the capabilities of modern object detection algorithms on datasets captured either in a low illuminance environment or in harsh conditions. In this paper, by taking a step forward in this direction, we propose a framework that leverages pixel-level information by employing the powerful recently proposed hybrid task cascade (HTC) network with a pre-trained ResNext-101 as a backbone network. The proposed pipeline is depicted in Figure 2.

To encapsulate, the main contributions of this work are explained below:

- This paper presents an end-to-end optimizable framework to tackle the problem of object detection under low illuminance and arduous conditions.
- We evaluated the proposed method on three different challenging datasets and achieved a mAP of 0.71, 0.52, and 0.43 on the datasets of ExDark, RESIDE, and CURE-TSD, respectively.
- Unlike previous works, the presented system does not rely on any domain-specific pre-processing techniques, such as image enhancement, to accomplish the results.

The remaining article is organized as follows. Section 2 describes the prior literature dealing with both generic and challenging environments through traditional computer vision or statistical learning-based approaches. Section 3 describes the presented object detection framework and the individual components. Section 4 presents the comprehensive overview of employed datasets. Section 5 explains the experimental details, evaluation metrics, and presents quantitative and qualitative analyses of the proposed system. Section 6 ends the paper with a brief conclusion and a discussion on the future work.

## 2. Related Work

Previous work in the field of object detection can be distinguished into two categories, namely generic object detection and object detection in a challenging environment [11]. Section 2.1 provides a brief overview of earlier approaches based on traditional computer vision algorithms to solve object detection in both generic and visually difficult environments. Section 2.2 discusses learning-based (mainly deep learning-based) methods in both environments.

### 2.1. Traditional Approaches

In the early days of computer vision [19], traditional algorithms used for object detection required handcrafted features and manual parameter tuning. Traditional algorithms can be categorized into approaches for the generic environment and the challenging environment.

#### 2.1.1. Generic Environment

The first traditional algorithm was the Viola–Jones (VJ) detector [20], which used a sliding window approach to find objects. Later, more advanced algorithms, such as the histogram of oriented gradients (HOG) detector [21] and the deformable part-based model [22], were introduced. Over the years, various surveys have been conducted on object detection in general environments [23,24,25,26], comparing different architectures from traditional to deep learning-based approaches, along with various datasets used as benchmarks to evaluate the performance of each algorithm [27].

#### 2.1.2. Challenging Environment

For challenging environments, traditional approaches for object detection employed template matching [28,29]. These approaches are difficult to extend to multiple classes, as for each object, a template is required. Later, Constantine et al. [30] proposed a method that uses wavelet representation with a support vector machine to detect objects in a given input image. The wavelet representation was calculated from statistical analysis of class instances. Another approach by Shirai et al. [31] for detecting objects required manual parameter tuning to find all objects and needed a few assumptions, such as the type and shape of an object, prior to detection.

### 2.2. Machine Learning-Based Approaches

Nowadays, deep learning-based algorithms are preferred as they automatically learn features and tune hyper parameters to find optimal results [32]. Similar to traditional approaches, learning-based approaches can be divided into two groups, learning-based approaches for generic environments and for challenging environments.

#### 2.2.1. Generic Environment

R-CNN [33] was the first learning-based network introduced in 2014 to solve the object detection problem. The network first extracted region proposals from the input image using selective search [34] and then combined them with convolutional neural networks (CNN) to find objects. In 2015, fast R-CNN [35] an improved version of R-CNN was proposed. Fast R-CNN passed the input image through CNN first to generate feature maps compared to its predecessor. Proposal regions were then selected from these generated feature maps using selective search. To take full advantage of resources, GoogleLeNet [36] was introduced after Fast R-CNN. Compared to the previous networks, GoogLeNet architecture allowed an increase in the width and depth of the network while keeping computation low. Compared to traditional algorithms, these networks performed better but still relied on selective search. Faster R-CNN [18] was the first network introduced that performed detection without relying on selective search. Faster R-CNN used a CNN network known as the region proposal network (RPN) [18] to find region proposals. In the year 2016, DenseNet [37] was introduced. DenseNet solved the vanishing-gradient problem and reduced the number of parameters required for training.

Later, mask R-CNN [38], an extension of faster R-CNN, was introduced. Mask R-CNN extended faster R-CNN [18] to pixel-level image segmentation by introducing an additional branch. Later in 2017, Retina-Net [39] was introduced, utilizing feature pyramid network(s) (FPN) [40] and focal loss to improve features and perform better detection. To solve the problem of overfitting, cascade R-CNN [41] was introduced. The cascaded architecture reduces the intersection over union (IoU) mismatches during training and inference time. Extending the network architecture of cascade R-CNN, hybrid task cascade [42] was introduced in 2019 with an additional branch for segmentation tasks. As backbones are essential components of object detection algorithms, several works recently proposed have improved the results over the years. One such example is Swin Transformer [43] introduced recently in 2021. The transformer-based architecture allows for greater efficiency by introducing a window-based self-attention mechanism and hierarchical feature map generation.

#### 2.2.2. Challenging Environment

Recent advancements in deep learning-based algorithms have given rise to various approaches to improve object detection in challenging environments [11]. Sasagawa et al. [14] proposed an approach to detect objects under low illumination by taking advantage of state-of-the-art algorithms and techniques of transfer learning. The idea is to combine two models from different domains with the help of a generative model and glue layers. Further, to train both models properly, the authors proposed using the knowledge distillation technique. First, spatial features are extracted from input by using an encoder–decoder network [44] composed of convolutional [45] and pooling layers [46]. With the help of pooling, layer features of different sizes and shapes are generated. The learned latent representation from the encoder–decoder network is propagated to the glue layer. After performing various experiments, the authors have established that the concatenation of all latent features produces the optimal result. After the glue layers, YOLO [47] is utilized to localize and identify objects. Another approach utilizing YOLO is proposed by Mate et al. [15] involving the use of thermal images instead of RGB images. As thermal images represent heat values, the authors establish that thermal images could improve object detection in low light environments and harsh weather conditions.

Another problem faced by object detection in a challenging environment is the loss of low-level features. Current object detection algorithms require high-level and low-level features to find objects and localize them [18]. The features help identify boundaries and different characteristics of objects present in the input image. These features are generally extracted from pre-trained backbones based on feature pyramid network (FPN) [40]. To preserve low-level features, Yuxuan et al. [48] propose the fusion of contextual information in the backbone. The fusion of features helps in maximizing pre-trained channel information. The second problem faced by object detection algorithms is that when images captured in low light are passed through conventional hierarchical convolutions, the resulting output contains shallow rich features. Therefore, context fusion is incorporated in the backbone part of the network, thus preserving information in features. At every stage, low-level feature maps of the network are selected and fused with their successor. The resulting feature map is then provided to the network to detect objects.

Following the introduction of two-stage detectors in object detection algorithms and the ability of generative adversarial networks to learn image transformations, the combination of formal and latter has been used to improve object detection performance. One approach by Kun et al. [16] involves combining deep convolution generative adversarial network(s) (DCGAN) [17] with faster R-CNN [18] to detect objects in low light. The combination of DCGAN and faster R-CNN involves three steps. First, DCGAN is used to learn and transfer the relationship between nighttime and daytime scenes. The second step is a multi-scale convolution feature fusion. Multi-scale convolutional feature fusion involves upsampling and downsampling of features to fuse them with their successors. The third step is to use an ROI pooling layer of different sizes to capture more detailed information. The authors argue that the standard ROI pooling layer reduces computational performance and loses the object’s critical features. Finally, ROI pooling output is given to faster R-CNN to obtain final results.

Another way of improving object detection is exploiting region-based convolutional neural networks, such as mask R-CNN [38] and instance segmentation approaches [49,50]. Avramovic et al. [51] proposed a method that uses selective parts of the input image to detect traffic signs in an arduous environment. As the driver only focuses on particular positions, such as the front mirror and back mirror, the authors argue that object detection should only be applied to those regions instead of the whole image. Selective object detection is performed by selecting a limited amount of regions of interest (RoIs), thus reducing the computational costs. The authors have evaluated their approach using mask R-CNN [38], and YOLO [47].

Kamal et al. [52] proposed integrating two different network architectures based on fully convolutional networks for semantic segmentation (FCNs) [53] to detect traffic signs. SegNet [54] and U-Net [55] are combined to detect signs, and a VGG-16 [56]-based network is used for classifying detected signs to their corresponding classes. SegNet and U-Net are trained by extracting corners of images and using them as training data. The resulting output of four patches is combined to create an output mask for the original image. The authors also used the L1 constraint term to modify Tversky loss [57] to increase the detection of small traffic signs.

In a challenging environment, generic object detectors predict multiple bounding boxes for a single object. Most of the generated bounding boxes have low confidence and can be removed with a non-maximum suppression technique [58], but not all overlapping detections are removed. To address this, Eran et al. [59] propose a Soft-IOU layer using Jaccard distance as a quality detector between the predicted bounding box and the ground truth. The second step of the proposed solution is to treat predictions from the network as a clustering problem. A custom EM-merger layer groups similar predictions into a single detection, thus removing overlapping detections. The authors performed various experiments on the SKU-110K dataset using Retina-Net [39].

Apart from object detection algorithms, semantic image segmentation (SIS) [60] has also been exploited to identify objects in arduous conditions. Unlike object detection algorithms, SIS tries to classify each pixel. Similarly, Ghose et al. [61] proposes a combination of saliency maps with thermal images to detect pedestrians in poor lighting conditions. Instead of using RGB and thermal images, the authors suggested that it is better to combine saliency maps and thermal images to find objects. First, thermal images are augmented with their corresponding saliency maps and are then provided to deep saliency networks. The combination helps illuminate salient parts of the image while preserving textural information, making it easier for the network to find objects.

Similar to previous approaches of combining thermal images with RGB images, Zhengzheng et al. [62] propose fusing RGB images with thermal images to detect objects in adverse conditions. A two-stream convolution neural network architecture generates features from RGB and thermal images. The output is fused to form a single feature representation. The authors argue that the fusion of features from RGB and thermal images helps preserve mid-level features, which are necessary for refining object details. A pyramid pooling module and a feature aggregation module to sharpen the object details are applied to the resulting features. The second contribution by the authors involves the use of a convolutional block attention module (CBAM) [63] to remove noise from features. CBAM is applied channel and spatial-wise. Finally, an average pooling layer is used to aggregate spatial information from features, and object detection is performed on them. The authors used a combination of edge and cross-entropy loss to train the proposed architecture.

## 3. Methods

### 3.1. Hybrid Task Cascade

Cascading has been used in computer vision for a long time [41]. It is a generic and dependable architecture that aids in improving performance. As a result, this design is employed to improve object detection performance. Iterative bounding box refinement [64] is a primitive approach for implementing cascading in object detection. There is an improvement in the performance of object detection. However, the improvement is not significant. Therefore, in object detection networks, a hybrid task cascade network presents a novel way of implementing the cascading design paradigm. To offer the spatial context, it first uses a fully convolutional branch. Second, it combines the detection and segmentation task within the cascade structure, allowing us to conduct both detection and segmentation at each level. As a result, we can name it collaborative multistage processing. Object detection and segmentation improve each other due to this cooperative multistage processing. Consequently, better detection can aid to enhance the performance of mask prediction and segmentation [42]. Figure 2 illustrates the proposed pipeline equipped with hybrid task cascade.

#### RCNN in Hybrid Task Cascade

The RCNN block is the fundamental block in a two-stage detector pipeline that computes the final task of localization and classification [18]. Cai et al. [41] enhanced this block by introducing cascading in which detection is refined under multiple stages. The HTC is built upon cascade R-CNN with a few modifications in our pipeline. The RCNN block is depicted in Figure 2. The proposals from the RPN are used as an input to the bounding box head (B1), after which the cascade begins, with each consecutive bounding box head receiving input from the corresponding ROI align. Each mask head receives an input, which is the fusion of semantic feature maps and the results of ROI align. The mask prediction head combines the two to produce accurate masks. In summary, RPN generates the first object proposals processed by ROI pooling. The initial bounding box coordinates are generated by the head B1 using the ROI pooling output. It also forecasts the object proposal’s confidence. In the second stage, M1 generates pixel-wise predictions in terms of masks. The other cascade levels follow the same pattern. In our proposed methods, we employ three stages with increasing IoU thresholds of 0.5, 0.6, and 0.7, respectively. The refined proposal features from the final stage are propagated to perform classification and regression.

### 3.2. Backbone Network

The backbone network is the fundamental part of the two-stage object detection methods since it extracts the spatial features and propagate the feature maps to the subsequent modules. In this paper, we utilize ResNeXt-101 [65] as the backbone network. The ResNeXt network extends the ResNet [66] architectures by providing the special cardinal features. A single layer of ResNeXt contains input channels, filter size, and output channels. This ResNeXt network has residual blocks. These residual blocks have two points: (i) the value of hyperparameters depends on spatial map size. (ii) If the spatial map size is reduced by 2, block-width becomes double. This provides uniform computation complexity.

In a neural network, neurons have aggregated-transformation in the form of inner product:

(1) $$\sum_{j=1}^{C}w_{j}n_{j}$$

where *n* is an input vector fed to the neurons having C-channels while $w_{j}$ is the weight of filter for *j*-th channel. The ResNeXt [65] also includes this type of transformation in a more specified form as a short network. The aggregated transformation equation is as given below:

(2) $$f\left(e\right)=\sum_{k=1}^{N}\tau_{k}\left(e\right)$$

where $\tau_{k}\left(e\right)$ can be a temporal function to place *e* into the lower-dimension and transform it, where *N* is the transformation size. The parameter *N* in Equation (2) is the same as C in Equation (1). However, these parameters are subject to change and can be tuned. The residual function can be mathematically explained as follows:

(3) $$Y_{out}=e+\sum_{i=1}^{D}\tau_{i}\left(e\right)$$

where $Y_{out}$ is the output function to be provided to the feature pyramid and region proposal network (RPN) of the employed HTC.

### 3.3. Feature Pyramid Network

After the backbone network, the second component of the two-stage detectors is a feature pyramid network (FPN) [40]. FPN is a feature extractor that takes a single-scale image of arbitrary size as input and outputs different sized feature maps at multiple levels in a fully convolutional fashion. The feature pyramid generated helps object detection network by providing features at different scales. FPN is usually applied after backbone operation and is independent of it. The bottom-up pathway is a feed-forward computation of a backbone consisting of features maps at several scales. The advantage of building a feature pyramid network is generating stable features captured at different scales from higher pyramid levels. The features are enhanced with features from the bottom-up pathway via lateral connections.

**Figure 3:**
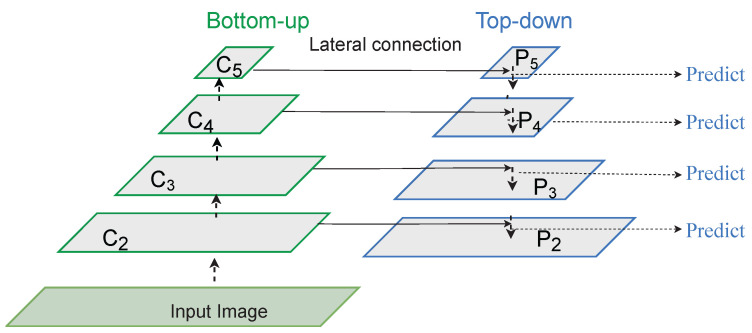
Visual illustration of the employed backbone and feature pyramid network. The backbone network extracts spatial features at multiple scales and propagates them to the corresponding FPN to learn various object representations.

In this work, we leverage the power of FPN by learning spatial features at different scales. Figure 3 illustrates the employed backbone and feature pyramid network in the proposed pipeline. The input image is passed through different resolutions where the scale on the upper level is reduced to half of the previous level. Each stage of the backbone communicates with the corresponding stage of FPN to enhance features at different scales. We employ four scales (P2, P3, P4, P5), receiving spatial features from corresponding stages of the backbone network of (C2, C3, C4, C5).

### 3.4. Region Proposal Network

Region proposal network (RPN) was introduced in faster R-CNN. Once features are generated from the feature pyramid or backbone network, the next step in a two-stage object detection network is to find the regions where the objects can exist. RPN can predict regions where objects can exist instead of looking at every pixel, thus reducing the computational cost. Before RPN can predict possible candidate regions, anchors are drawn. Anchors are bounding boxes drawn with various sizes and scales on feature maps and represent the objects that networks need to detect. The size and shape of anchors can be configured from the dataset.

The RPN network is composed of CNN layers and has a classifier and a regressor. The classifier part determines the probability of a proposal having the target object, and the regressor part regresses the coordinates of the proposal. RPN operates similar to any other CNN network by sliding a window over the features and predicting whether the anchors drawn in the region contain an object or not. Only the anchors with the highest IoU are assigned labels and used in later stages. RPN is trained along with other components of two-stage detectors during training. The loss function of RPN network is illustrated in Equation (4) as:

(4) $$L\left(p_{i},t_{i}\right)=\left(1/N_{cls}\right)\times \sum L_{cls}\left(p_{i},p_{i}^{*}\right)+\left(\gamma /N_{reg}\right)\times \sum p_{i}^{*}L_{reg}\left(t_{j},tj^{*}\right)$$

where *i* donates the anchor index in a batch, and $p_{i}$ denotes the probability that an anchor is an object or not. Ground truth $p_{i}^{*}$ is 1 if the anchor is positive and is 0 if the anchor is negative. Similarly, $t_{i}$ denotes the vector of 4 parameterized coordinates of the predicted bounding box, and $t_{i}^{*}$ represents the ground truth box. The classification loss $L_{cls}$ is log loss over two classes (object vs non-object). For the regression $L_{reg}$, the loss function is shown in Equation (5) as:

(5) $$L_{reg}\left(t_{i},t_{i}^{*}\right)=R\left(t_{i}-t_{i}^{*}\right)$$

where *R* is robust loss function (smooth L1) defined in [35], $t_{i}$ represents ground truth box and $t_{i}^{*}$ represents predicted bounding box. The term $N_{cls}$ represents the normalization factor for classification loss and is equal to the batch size. The term $N_{reg}$ represents the normalization factor regression loss and is equal to the number of anchor locations. γ is used for balancing parameters and, by default, is set to 10 unless stated otherwise. In our experiments, we set a single scale of RPN to 8 with three ratios [0.5, 1.0, 2.0] and five different strides of [4, 8, 16, 32, 64].

## 4. Datasets

### 4.1. ExDark

**Figure 4:**
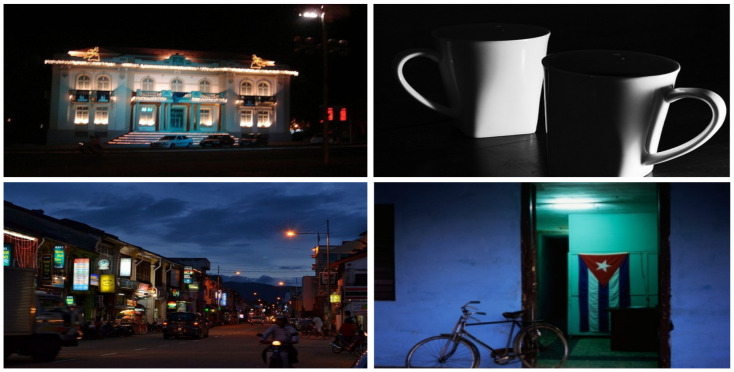
Samples taken from ExDARK dataset. The dataset has images captured in low light and indoor scenes.

One of the most challenging and openly available datasets is the ExDARK [13] dataset created in 2020. The dataset comprises 7363 low-light pictures captured in different indoor and outdoor environments at nighttime. There is a total of 12 classes in the dataset. For the sake of variety, image enhancement techniques, such as de-hazing and blurring, as augmentations are applied. The dataset contains the following classes: table, cat, people, motorbike, dog, cup, chair, bicycle, boat, bottle, bus, car, and cat. Figure 4 exhibits few samples from this dataset.

### 4.2. CURE-TSD

**Figure 5:**
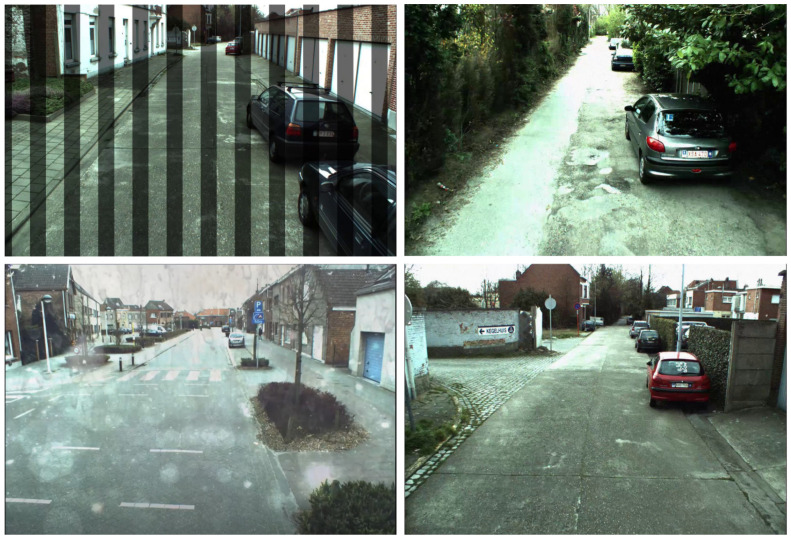
Dataset samples taken from CURE-TSD. Heavy augmentation is applied to increase challenge for object detection algorithms.

CURE-TSD [67] is a large challenging dataset for the task of traffic sign detection. The dataset is composed of videos captured by driving a car around at different times of the day. Different augmentations, such as decolorization, blur, darkening, dirty lens, exposure, codex error, snow, and haze, are applied to introduce variety. There are 14 types of traffic signs in this dataset: speed limit, goods vehicles, no overtaking, no stopping, no parking, stop, bicycle, hump, no left, no right, priority to, no entry, yield, parking. Figure 5 illustrates few samples of this dataset.

### 4.3. RESIDE

**Figure 6:**
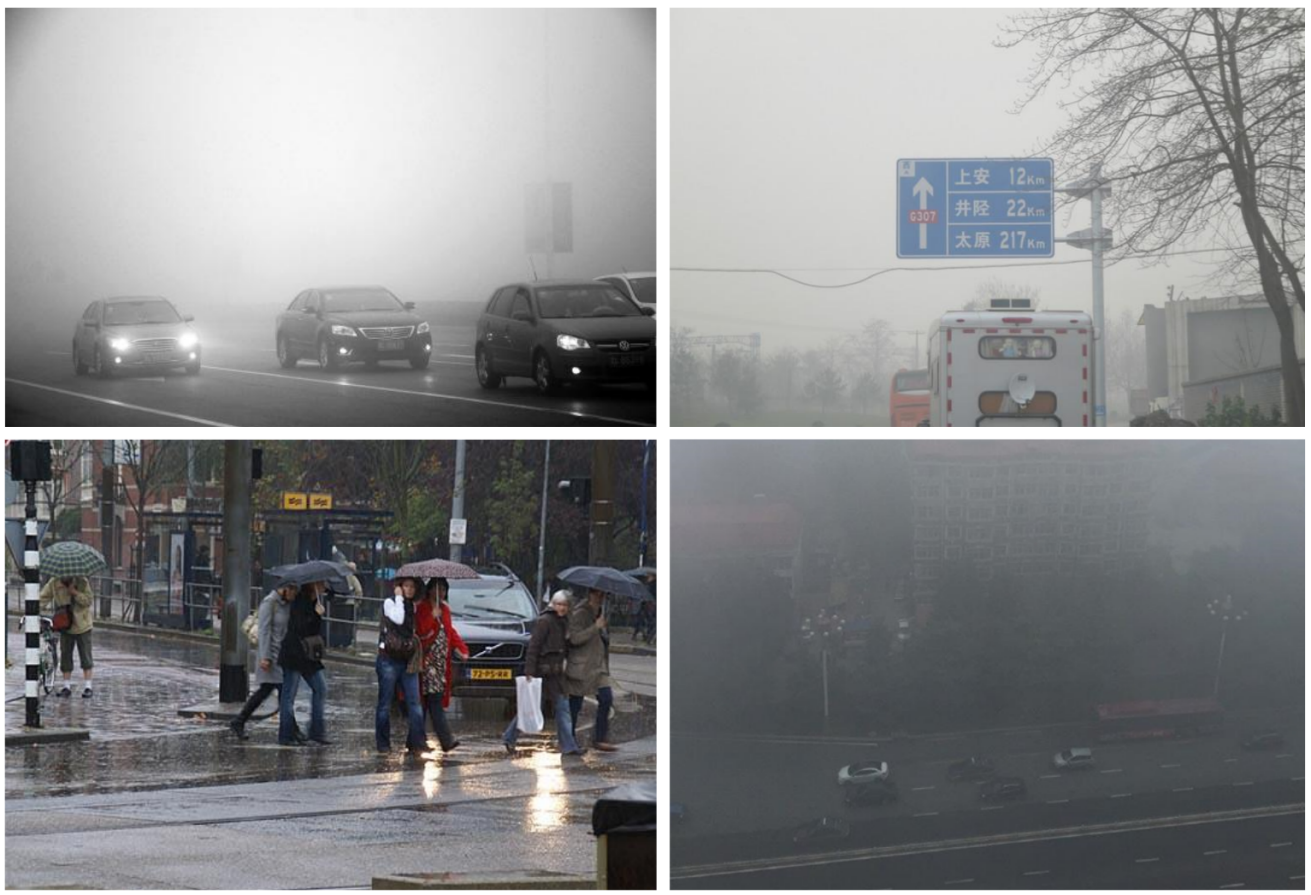
Dataset sample taken from RESIDE dataset. This dataset contains images of vehicles, roads, traffic signs, and signboards explaining directions captured in harsh weather conditions.

Another challenging dataset employed in our approach is RESIDE dataset [68]. The dataset is mainly for the task of object detection in difficult weather. The subset RTTS comprises 4332 real-world hazy images representing different scenarios in a day. Images are collected manually through video cameras and annotated with bounding boxes localizing objects. The dataset contains various real-world occlusions, such as hazy, rainy, and snowy weather. There are five annotated object classes in the dataset as bicycle, bus, motorbike, car, and person. Figure 6 depicts few samples from this dataset.

## 5. Experimental Results

### 5.1. Implementation Details

The codebase of the presented system is based on the MMDetection framework [69]. The backbone network is ResNext-101, which is pre-trained on ImageNet [45]. The cardinality of the backbone network is set to 64, and the bottleneck width is defined as four unless stated otherwise. We train on all three datasets with identical configurations. All datasets are fine-tuned for ten epochs, with a learning rate of 0.0025. SGD is used as an optimizer with a batch size of 4 on a single GPU machine. There are no augmentations applied during pre-processing, and only random horizontal flip is applied. Image sizes are kept variable in the range of 800 × 1388 while maintaining their aspect ratio.

### 5.2. Evaluation Protocol

Considering the problem of object detection in a challenging environment is identical to generic object detection, we evaluate our method on similar evaluation metrics:

#### 5.2.1. Precision

Precision [70] computes the ratio between the predicted samples present in ground truth and the total predicted samples. Mathematically, it is explained below:

(6) $$\frac{\mathrm{Predicted}\;\mathrm{samples}\;\mathrm{in}\;\mathrm{ground}\;\mathrm{truth}}{\mathrm{Total}\;\mathrm{predicted}\;\mathrm{samples}}=\frac{\mathrm{TP}}{\mathrm{TP}\;+\;\mathrm{FP}}$$

where TP denotes true positives and FP represents false positives.

#### 5.2.2. Recall

Recall [70] is the ratio between correctly predicted samples and total samples present in ground truth. The formula for the recall is given by:

(7) $$\frac{\mathrm{Predicted}\;\mathrm{samples}\;\mathrm{in}\;\mathrm{ground}\;\mathrm{truth}}{\mathrm{Total}\;\mathrm{samples}\;\mathrm{in}\;\mathrm{ground}\;\mathrm{truth}\;\mathrm{region}}=\frac{\mathrm{TP}}{\mathrm{TP}\;+\;\mathrm{FN}}$$

where TP is true positives and FN represents false negatives.

#### 5.2.3. Average Precision

Average precision (AP) is defined as the weighted sum of precision at different IoU thresholds and the weight represents the change in the value of a recall. The formula for calculating average precision is mathematically expressed as follows:

(8) $$\mathrm{AP}=\sum_{n}\left(R_{n}-R_{n-1}\right)P_{n}$$

where $R_{n}$ and $P_{n}$ are the precision and recall at the *n*_th_ threshold.

#### 5.2.4. Intersection over Union

Intersection over union (IOU) [71] defines the amount of predicted area intersecting with the actual ground truth area. Mathematically, IOU is given by:

(9) $$\mathrm{IOU}=\frac{\mathrm{Area}\;\mathrm{of}\;\mathrm{intersection}\;\mathrm{between}\;\mathrm{prediction}\;\mathrm{and}\;\mathrm{ground}\;\mathrm{truth}}{\mathrm{Area}\;\mathrm{of}\;\mathrm{Union}\;\mathrm{between}\;\mathrm{prediction}\;\mathrm{and}\;\mathrm{ground}\;\mathrm{truth}}$$

#### 5.2.5. Mean Average Precision

Mean average precision (mAP) is an important evaluation metric for category-specific performance. The mAP can be computed by simply taking the mean of AP achieved in each class. The formula for mAP is explained as follows:

(10) $$\mathrm{mAP}=\frac{1}{N}\sum_{i=1}^{N}AP_{i}$$

where *N* represents total classes and $AP_{i}$ is the average precision for a given class.

### 5.3. Result and Discussion

To assess the capabilities of the proposed method, we evaluate the proposed system on three publicly available challenging datasets. This section discusses the results achieved on all of three datasets.

#### 5.3.1. ExDark

We validate the performance of our system on the challenging ExDark dataset [13]. Table 1 presents the quantitative analysis of the proposed method. Moreover, it compares our results with previous state-of-the-art methods. Our method surpasses the previous state-of-the-art results with an mAP of 0.71 on a varying IoU threshold from 0.5–0.95. On the IoU threshold of 0.5, our method achieves an AP of 0.94.

**Figure 7:**
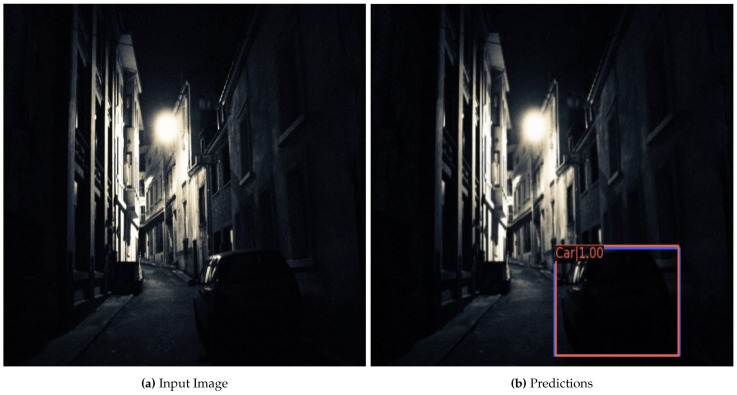
Example of results achieved on the ExDark Dataset. (**a**) represents an input image, whereas (**b**) is the final output with the detected object. The blue color represents ground truth annotation, and orange is the network prediction.

The promising results on the low illuminance dataset illustrate that the extra segmentation module present in the employed HTC network facilitates the network to detect objects even in darker conditions. For complete understanding, Figure 7 depicts an instance of localizing and classifying a car in a dark image. Although the car is difficult to detect with a naked eye, our system detects it with a confidence of 100%.

##### Comparison with State-of-the-Art Methods

By looking at Table 1, it is evident that our approach beats the prior best results with a mAP difference of four points. The previous best results were achieved by Ahmed et al. [11] with a mAP of 0.67, and Loh et al. [13] by achieving a mAP of 0.49.

#### 5.3.2. RESIDE

Analogous to ExDark, we report the performance on the RESIDE dataset, which is explained in Section 4.3. By analyzing Table 2, one can observe that the proposed method can further enhance the performance of object detection on the challenging RESIDE dataset. On an IoU threshold range from 0.5 to 0.95, we achieve a mAP of 0.52, whereas the AP of the proposed system goes to 0.81 on an IoU threshold of 0.5.

**Figure 8:**
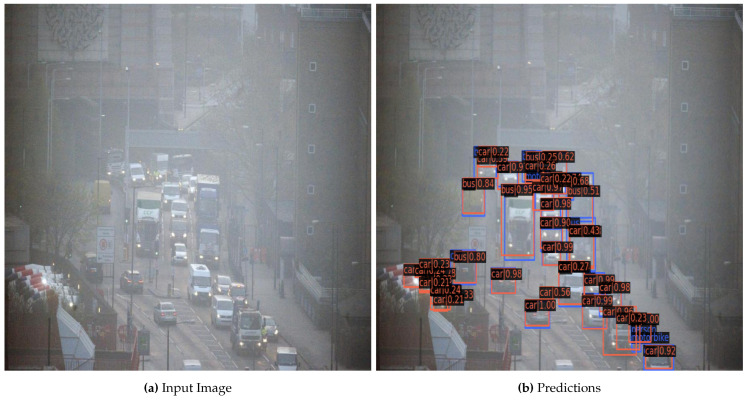
Example of results achieved on the RESIDE Dataset. (**a**) represents an input image, whereas (**b**) is the final output with the detected object. The blue color represents ground truth annotation, and orange is the network prediction.

Figure 8 exhibits the qualitative performance of the system. In Figure 8a, it can be seen that the image is visually challenging to interpret and Figure 8b shows the capabilities of the method to detect several objects present in the ground truth. However, on the left part of Figure 8b, one can observe a few instances of false positives with lower confidence scores.

##### Comparison with State-of-the-Art Methods

As summarized in Table 2, the previous best results obtained on the RESIDE dataset were achieved by Ahmed et al. [11] with a mAP of 0.51. The proposed method in this paper pushes the previous state-of-the-art to the new best score of 0.52.

#### 5.3.3. CURE-TSD

CURE-TSD is the last dataset in which we assess the capabilities of the presented work. Table 3 presents the results achieved by our method on the CURE-TSD dataset. We achieve an mAP of 0.43 on an IoU threshold ranging from 0.5 to 0.95, whereas we attain an AP of 0.55 on an IoU threshold of 0.5. Furthermore, we achieve an AP of 0.06, 0.23, and 0.34 on the smaller, medium, and larger objects, respectively.

**Figure 9:**
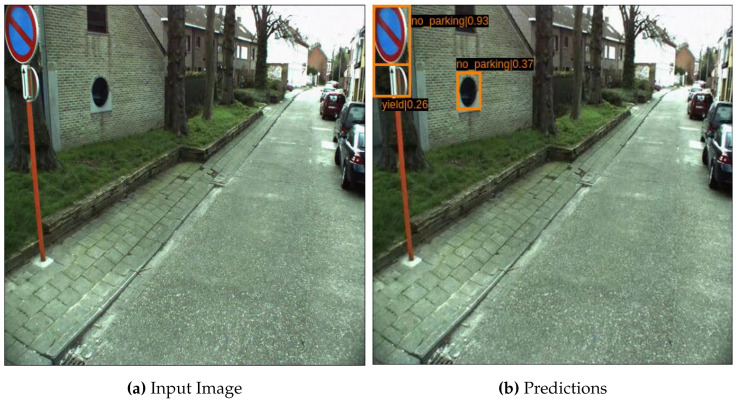
Example of results achieved on the CURE-TSD Dataset. (**a**) represents an input image, whereas (**b**) is the final output with the detected object. The blue color represents ground truth annotation, and orange is the network prediction.

The qualitative analysis of our method is illustrated in Figure 9. In the mentioned figure, it can be perceived that the network has successfully detected a stop sign. However, owing to the high inter-class variance with other objects, the network produces a couple of false positives. Furthermore, the network produces a false positive by detecting a sign on the wall that appears similar to other objects in the dataset. This result raises an interesting question of how much prior context can improve this result [73].

##### Comparison with State-of-the-Art Methods

By looking at Table 3, the previous best mAP attained on the CURE-TSD dataset is attained by Ahmed et al. [11] with a mAP of 0.28. However, the presented system outsmarts the prior results with a mAP of 0.43. Moreover, we observe a noticeable increase in the AP achieved on an IoU threshold of 0.5. It is essential to mention that Kamal et al. [52] achieved an AP of 0.94. However, we were unable to find the mAP score in the paper. Therefore, our results are not directly comparable with [52].

#### 5.3.4. Effect on Increasing IoU Thresholds

**Figure 10:**
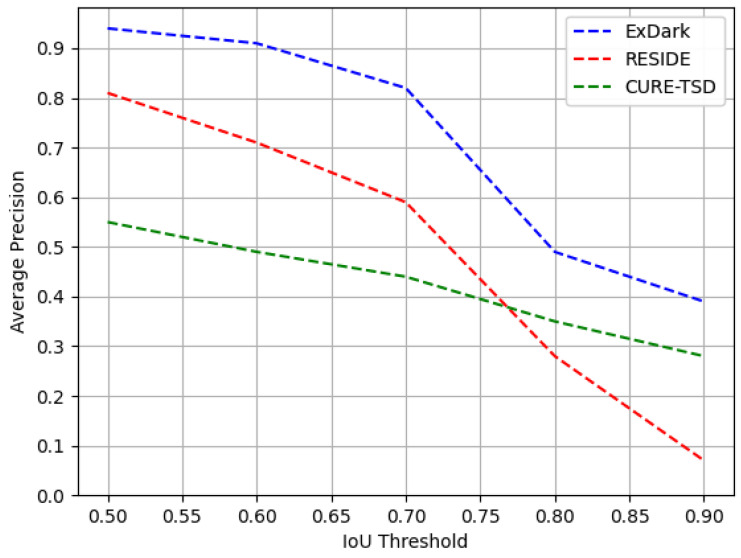
Performance analysis of the proposed module on increasing IoU thresholds from 0.5 to 0.9 on all three employed datasets.

In order to assess the robustness of the proposed method, we evaluate the trained models on varying IoU thresholds on their respective test sets. Figure 10 exhibits the performance in terms of AP computed on an increasing IoU threshold from 0.5 to 0.9. It is evident that due to the incorporation of pixel-level information, the proposed method is capable of producing satisfactory results even on higher IoU thresholds of 0.7. The performance drops drastically upon increasing the IoU threshold further from 0.7. These results exhibit room for improvement by designing more efficient and robust detection methods in future.

#### 5.3.5. Effect with Different Backbone Networks

For completeness, we further conduct experiments on the ExDark dataset by employing different backbone networks. The purpose of these experiments is to assess the role of the proposed pixel-level method and backbone networks in yielding superior results. Table 4 presents a comprehensive summary of our proposed method equipped with three different backbone networks. It is evident that even with the relatively smaller backbone of ResNet-50 [66], the proposed method surpasses the performance of the previous best results by Ahmed et al. [11] (see Table 1).

##### Performance against Computation

It is essential to demonstrate the relative comparison between the increase in performance with the increase in computational capacity and real-time. By looking at Table 4, we observe that upon employing ResNet-101 as a backbone network, we experience a slight boost from 0.68 to 0.69 in mAP and a reduction in the run-time from 5.8 to 5.5 FPS. Furthermore, the mAP increases to 0.71 with ResNext-101 with a slight further decrease of 5.0 FPS. The best trade-off between performance and computation is achieved with ResNext-101 with a mAP of 0.71 and FPS of 5.0. We believe that this work will motivate future research to present the trade-off between performance gains and computations.

## 6. Conclusions and Future Work

This research proposes an end-to-end optimizable system for tackling the challenge of object recognition in low-light and difficult environments. The proposed approach utilizes a hybrid task cascade network to effectively exploit pixel-level information at different cascade levels. On the ExDark, RESIDE, and CURE-TSD datasets, we have mAPs of 0.71, 0.52, and 0.43, respectively, by evaluating the suggested technique on three different challenging datasets. Unlike prior efforts, the presented method achieves its outcomes without pre-processing techniques, such as picture augmentation. In the future, we plan to apply the idea of exploiting pixel-level information on other challenging datasets [59,74,75]. Furthermore, an end-to-end trainable pixel-level enhancement and learning approach would be another interesting future direction. Moreover, we aim to design robust detection methods that yield real-time performance by extending the proposed direction in the future.

## Figures and Tables

**Table 1:** Comparison between the proposed method and previous state-of-the-art results on the ExDark dataset. AP_s_ denotes the average precision for a small area, whereas AP_m_ represents the average precision for a medium area and AP_l_ depicts the average precision for a large area. The IoU threshold is also defined in the table. The best results are in bold.

Methods	mAP(0.50:0.95)	AP^50^(0.50)	AP_s_(0.50:0.95)	AP_m_(0.50:0.95)	AP_l_(0.50:0.95)
Ahmed et al. [11]	0.67	0.93	0.50	0.61	0.71
Yuxuan et al. [48]	0.34	0.64	0.03	0.17	0.40
Loh et al. [13]	0.49	0.79	-	-	0.53
Chen et al. [72]	0.32	-	-	-	-
Our Method	**0.71**	**0.94**	**0.57**	**0.69**	**0.75**

**Table 2:** Comparison between the proposed method and previous state-of-the-art results on the RESIDE dataset. AP_s_ denotes average precision for the small area, whereas AP_m_ represents the average precision for the medium area and AP_l_ depicts the average precision for the large area. The IoU threshold is also defined in the table. The best results are in bold.

Methods	mAP(0.50:0.95)	AP^50^(0.50)	AP_s_(0.50:0.95)	AP_m_(0.50:0.95)	AP_l_(0.50:0.95)
Ahmed et al. [11]	0.51	0.79	**0.40**	0.11	0.57
Our Method	**0.52**	**0.81**	0.26	**0.40**	**0.57**

**Table 3:** Comparison between the proposed method and previous state-of-the-art results on the CURE-TSD dataset. AP_s_ denotes the average precision for the small area, whereas AP_m_ represents the average precision for the medium area, and AP_l_ depicts the average precision for the large area. The IoU threshold is also defined in the table. The best results are in bold.

Methods	mAP(0.50:0.95)	AP^50^(0.50)	AP_s_(0.50:0.95)	AP_m_(0.50:0.95)	AP_l_(0.50:0.95)
Ahmed et al. [11]	0.28	0.38	0.06	0.23	0.34
Kamal et al. [52]	-	**0.94**	-	-	-
Our Method	**0.43**	0.55	**0.12**	**0.26**	**0.53**

**Table 4:** Summarizing the trade-off between performance and efficiency of the proposed method with different backbone networks on the ExDark dataset.

Backbone Network	mAP(0.50:0.95)	AP^50^(0.50)	Memory (GB)	FPS
ResNet-50+FPN	0.68	0.93	8.2	5.8
ResNet-101+FPN	0.69	0.94	10.2	5.5
ResNeXt-101+FPN	0.71	0.94	11.4	5.0

## Data Availability

Publicly available datasets are employed in this study for experiments. These data can be found here: https://github.com/cs-chan/Exclusively-Dark-Image-Dataset (accessed on 28 April 2022).
